# Bibliographical Notices

**Published:** 1847-08

**Authors:** 


### BIBLIOGRAPHICAL NOTICES.

SYDENHAM SOCIETY. *The Works of William Harvey, M. D., Physician to the King, Professor of Anatomy and Surgery to the College of Physicians. Translated from the Latin, with a Life of the Author.* By ROBERT WILLIS, M. D., Member of the Royal College of Physicians and Surgeons of England, Corresponding member of the Royal Academy of Sciences of Göttingen, &c. 8vo. pp. 624. London, 1847.

It was but right, that one of the earliest labours of the Sydenham Society should be directed to the preparation of a complete edition of the works of the great Englishman, whose discovery of the complete circulation of the blood has immortalized his name; for whatever may be the disposition—and a proper one it is—to do justice to the predecessors of Harvey, it is pretty universally admitted, notwithstanding the views and arguments adduced by our learned and venerable friend, Dr. Cox, that the perfection of the great discovery was his; and when, it may be asked, has a discovery of the kind ever been made *de novo*? Observation after observation has usually been registered for a long period; until ultimately some one has topped the pyramid, and completed the undertaking; whilst, as in the case of every great discovery or invention, the surprise has been, that so apparently simple a result should not have been attained by hundreds as well as by the 'lucky' individual who made the last and most important step.

It was not—as we have previously said, when noticing this same subject—the person who first discovered the propulsive power of steam, or even he who first rudely applied it to the formation of an imperfect engine, that deserved the credit of that wonderful result of human ingenuity—the steam engine; but the man who, like Watt, made it a master-piece of mechanism; or, like Fulton, showed that it could be applied so wonderfully and fearfully to the purposes of navigation.

The editor of the edition of Harvey's works, now before us, with much self-complacency—which is, indeed, too apparent throughout his preface—flatters himself, that he has "set his [Harvey's] claims to the whole and sole merit of the discovery of the circulation in a new and clearer light than they have yet been seen;" and that he has done "more than any preceding biographer in exhibiting his moral nature; for truly he was as noble in nature as he was intellectually great." p. viii.

Dr. Willis gives, at considerable length, the views of the predecessors of Harvey, as well as of his opponents—contemporaneous and others—on the great question of the circulation. His predecessors, doubtless, paved the way for the ultimate discovery; and we would pause, therefore, before assenting to Dr. Willis's conclusion, that Harvey has "claims to the whole and sole merit of the discovery of the circulation." Indeed, Dr. Willis is scarcely consistent on this point, for in alluding to the views of Fabricius of Acquapendente, who had given especial attention to the valves of the veins, he adds:

"Fabricius could observe, and he could describe; but he wanted the combining intellect that infers, the imagination that leads to new ideas—to discovery. Though he did little himself, however, to advance the sum of human knowledge, he proved a tooth in the wheel that has since put in motion the whole machinery of medical science (?). He it was who sowed the seed, little dreaming of its kind, which, finding one spot of congenial soil, sprang up a harvest that has continued to nurture the world of physiological science to the present hour."

We have looked carefully over the remarks of Dr. Willis on this formerly much contested subject: but we confess, with every disposition to render him ample justice, we cannot arrive at the conclusion, that he has "set Harvey's claims to the whole and sole merit of the discovery of the circulation in a *new* and *still clearer* light than they have yet been seen." We are unable to discover the *new*, and it is not a more easy matter to detect the *still clearer*, for which he asks credit. The "excellent summary"—as he properly terms it—of the entire doctrine of the circulation by Dr. Freind in his Harveian oration, appears to us more novel, and at least as clear. To this Dr. Willis refers his reader for other information—adding, neither in the proper spirit of courtesy nor of philosophy—

"I pass by the s'ill-recurring denials by obtuse and ill-informed individuals, of the truth, or of the sufficiency of the evidence of the truth, of the Harveian circulation. Those who *can* not see, must, contrary to the popular adage, be admitted to be still blinder than those who *will* not see."

The following is Dr. Willis's conclusion :

"Having now disposed of the claims that have been set up in behalf of one or another, as the discoverer of the circulation, and shown, we trust satisfactorily, that these are alike untenable, we should now proceed to discuss the question of cui bono?—but this meets us in so forbidding an aspect, brimful as is our mind with a sense of the all-importance of the knowledge we had from Harvey, and seems so little to belong to our subject, that we gladly pass it by unnoticed ; though it be only to find ourselves encountered by that other topic, but little more congenial to our mood of mind and intimate persuasion—the merit of Harvey as a discoverer. Few, very few, have been found to question this ; but as one man of undeniable learning and eminence in his profession,* has very strongly, it seems to us, been led to do so, it will not be impertinent if we cast away a few words on this matter.

Discovery is of several, particularly of two kinds ; one sensible or perceptive ; the other rational or inductive ; the former an act of simple consciousness through an impression made on one or more of the senses ; the latter a conclusion come to by the higher powers of the understanding dealing with data previously acquired by the senses and perceptive faculties. We look through a telescope, for example, and we perceive a star which no one had seen before ; we note the fact, and so become discovers of a new star. The merit here is not, surely, very great, though the added fact may be highly important. Again ; one of the planets is subject to such perturbations in its course that to compose exact tables of its orbit is held impossible. These perturbations are referable to none of the known perturbating causes. A great astronomer suggests the influence of an exterior and unknown planet as their cause. A consummate mathematician and

* Dr. William Hunter. Introductory Lectures, p. 59, (4to. London, 1784,) to which the reader is referred for a singularly inconsistent and extraordinary string of passages.

physical astronomer makes trial of this suggestion: he assumes the ascertained perturbations as elements; he combines these under the guidance of knowledge and reason, and at length he says, that if the course suggested be well founded, there or thereabouts it must exist; and lo! on turning the far-seeing tube to the point in space he had indicated, there in verity gleams a new world, then first seen, though launched by God from eternity to circle on the verge of our creation; and he who bade us look, became the discoverer of a new planet. Who will dispute the merit here? Truly, man does show the God within him when he uses his faculties—God-like in themselves—in such God-like fashion. But Harvey's merit, according to our idea, was of the self-same description in another sphere. The facts he used were familiarly known, most of them to his predecessors for nearly a century, all of them to his teachers and immediate contemporaries; yet did no one, mastering these facts in their connection and sequence, rising superior to prejudice, groundless hypothesis and erroneous reasoning, draw the inference which now meets the world as irresistible, until the combined mind of Harvey gave it shape and utterance. To our apprehension Harvey was as far above his fellows, as the eye of poetic intelligence, which exultingly absorbs the beauties of the starry sky, and the green earth, is above the mere physical sense which distinguishes light from dark. The late Dr. Barclay, a fervent admirer of Harvey, whose name he never uttered without the epithet immortal, has put the question of Harvey's merit both happily and eloquently, and it affords us pleasure to quote the passage from the writings of our old and honoured teacher in anatomy. "The late Dr. Hunter," says Dr. Barclay,* "has rather invidiously introduced Harvey along with Copernicus and Columbus, to show that his merit as a discoverer was comparatively low. But what did Copernicus, and what did Columbus? Not in possession of more numerous facts than their contemporaries, but endowed with nobler and more vigorous intellects, the one developed the intricate system of the heavenly bodies, and the other discovered an unheard of continent. Was it not in the same way, by the exertion of superior intellect, that Harvey made his immortal discovery? I know not what has happened in the world unseen; but if I may judge from the records of history and the annals of fame, the spirit of Bacon, the spirits of Columbus, Copernicus and Newton, have not been ashamed to welcome and associate with the congenial spirit of Harvey." To this fine passage there is little to be added: Harvey's discovery

* On the Arteries, Introduction, p. 9.

was of the rational and inductive, and therefore higher class, according to our estimate; it was made in virtue of the intellectual powers which peculiarly distinguish man in a state of the highest perfection."

The volume before us, after the Preface, Life of William Harvey, and his last will and testament, contains "An anatomical disquisition on the motion of the heart and blood of animals;"—"The first anatomical disquisition on the circulation of the blood, addressed to John Riolan;"—"A second disquisition to John Riolan; in which many objections to the circulation of the blood are refuted;"—"Anatomical exercises on the generation of animals; to which are added essays on parturition; on the membranes and fluids of the uterus, and on conception;—Anatomical examination of the body of Thomas Parr; concluding with sundry letters on matters of science, addressed to Dr. Caspar Hofmann, Paul Marquerd Slegel, John Nardi, R. Morison, John Daniel Horst and others."

The works of Harvey were written originally in Latin; but most of them were rendered into English; so imperfectly, however, in some cases, that Dr. Willis says he undertook the task of translating them anew. The "masterwork" of Harvey on the motion of the heart and blood, he found to "have been translated by one but little conversant with the subject," and "that it was both extremely rebutting (?) in point of style, and full of egregious errors," so that "nothing short of an entirely new translation could do justice to this admirable treatise, or secure for it, at the present day, the attention it deserved."

The work on generation he likewise translated over again, as well as that on the anatomy of Parr. The letters have never appeared in English before.

We have not had time to compare Dr. Willis's version with the original, in order to decide whether he has avoided the charges he has laid against his predecessors. It becomes him, however, to have been extremely cautious; and we cannot avoid thinking that the fact of his having entirely retranslated works which had been generally received as correct versions of the original, savours not a little of the self-complacency to which we have before alluded; as well as a desire to absorb the whole credit;—seeing that it would have been easy for him to have compared the original with the translation, and to have corrected anything "rebutting" in the style; or any inaccuracy in conveying the meaning of the author; and we must say, in conclusion, that whilst we hail with unallayed pleasure this edition of the works of one of the shining lights of medical science, we are by no means satisfied that the best possible selection has been made of Editor.

---

*Observations on Aneurism, and its Treatment by Compression.* By O'Bryen Bellingham, M. D., Edin. Fellow of and Professor in the School of the Royal College of Surgeons in Ireland, etc. etc. etc. John Churchill. London: 1847.

In this interesting *brochure*, Dr. Bellingham gives us a brief history of the treatment of Aneurism by Compression, and the various improvements in the mode, accompanied by an abstract of the cases that have been reported in which compression has been used; the instruments employed, and the theories upon which compression has been supposed by different writers to cure aneurism.

"The author's views respecting the pathology of aneurism, particularly the exact manner in which the disease is cured, differ from those usually taught;" and he has endeavoured to prove that "the cure of aneurism is accomplished in a similar manner, whether the ligature is employed or compression is used, or whether the cure has been brought about by Nature's unaided efforts." That the latter is true, is highly probable, from the uniform simplicity observed in the processes of Nature on other occasions, and the explanation of our author, which we shall notice presently, is at least plausible. The employment of cutting instruments upon the living body, where it can be avoided without incurring other or greater evils, is always to be deprecated, and every plan which holds forth a reasonable hope of relief without such a resort, is entitled to calm and impartial consideration. The result of all the experiments for the cure of aneurism by compression, has not been such as to warrant our confidence in relying upon it in the generality of cases; but if it can be shown that a false theory has been entertained on this subject, under the guidance of which an erroneous practice has been pursued, the case becomes altered in its aspect most essentially.

The objections that have been urged against compression by authoritative writers are principally the following:

1st. Uncertainty of success, from the impossibility of applying sufficient force to bring the sides of the vessel into contact, so as to cause adhesive inflammation and consequent obliteration of its cavity, in many instances from its being surrounded by soft and elastic tissues that take off the pressure.

2d. Impossibility of continuing the pressure long enough to be effectual, in consequence of the extreme suffering of the patient from compression of the large nerves which accompany all the principal arteries.

3d. Danger of sloughing, in consequence of the long continued pressure.

All these evils have undoubtedly been experienced, and the question is, whether, according to Dr. Bellingham's theory, and the practice founded upon it, success can be attained without such risks.

"Surgical writers appear," says Dr. B., "to have been under the impression, that in order to cure an aneurism by compressing the artery above the tumour, it was essential to interrupt completely the current of blood through the vessel—in fact, to to apply such pressure as would act like a ligature, cause inflammation of the coats of the artery at the part, and obliterate the circulation in the vessel at the point to which compression had been applied.

"When it was considered absolutely necessary for the success of compression, that such an amount of pressure should be applied as was almost certain to produce sloughing of the part, and very certain to occasion intense pain and suffering; and when, in addition, this was to be prolonged through five successive nights and days, (as in the case reported by Mr. Guthrie) we can readily understand why patients refused to submit to it, and we can easily account for the disrepute in which the practice fell; and for the unwillingness of surgeons to adopt this treatment, in preference to the simple operation of placing a ligature upon the femoral artery. It would, however, appear that it is not at all essential that the circulation through the vessel leading to the aneurism should be completely checked, but rather the contrary: it may, perhaps, be advantageous at first, for a short period, by which the collateral circulation will be more certainly established; but the result of this case, if it does no more, establishes the fact, that *a partial current through an aneurismal sac will lead to the deposition of fibrine in its interior, and cause it within a few hours to be filled and obstructed, so as no longer to permit of the passage of blood through it.*

Pressure, so as altogether to obstruct the circulation in an artery, must necessarily be slower in curing an aneurism, as it must, in some measure, act by causing obliteration of the vessel at the part to which the pressure has been applied; whereas *a partial current through the sac enables the fibrine to be readily entangled in the parietes of the sac in the first instance, and this goes on increasing until it becomes filled;* the collateral branches having been previously enlarged, the circulation is readily carried on through them."

Several cases are narrated by the author, wherein these results of moderate compression were proved to have taken place, by post mortem inspection of the parts.

That aneurism may be cured in the manner recommended by Dr. Bellingham is admitted by eminent living surgeons, but the priority of the practice as well as the theory is not so readily accorded to him. Mr. Wilde, the able editor of the Dublin Quarterly Journal, awards the credit to the late Mr. Todd of that city. Professor Syme of Edinburgh, whilst he denies to Dr. B. any just claims to originality, condemns the practice as inapplicable to aneurism of the large arteries generally, although he admits that, in certain cases, where for special reasons the ligature cannot be used, compression should be tried.

If the doctrine contended for, however, and which it is not difficult to test, shall be established by sufficient observation, we can see no reason why compression should not supersede the knife and the ligature in nearly all cases, and should Dr. Bellingham's earnest advocacy be productive of so good and desirable an improvement, he will indeed have earned a proud claim to our gratitude.

The following remarks, extracted from Chapter X., present a *resumè* of the positions assumed by Dr. B.:—

"When we consider how many writers have devoted their attention almost exclusively to the subject of aneurism, and how much talent has been engaged in illustrating its history, pathology, and treatment, it appears strange that the process which nature herself sets up for its cure should have been so much overlooked hitherto by surgeons; and although this process was daily, I may say, passing under their eyes, that the exact mode in which it was accomplished should have attracted but little attention, and no attempts should have been made to imitate or assist it.

In almost every case of aneurism, where the disease has subsisted for some time, we find a larger or smaller amount of solid matter deposited in the sac, which is composed of the fibrine of the blood, arranged in regular concentric laminæ.

Examples of the spontaneous cure of aneurism, in which the sac is completely filled with fibrine deposited in regular concentric layers, are not very uncommon.

Again, in cases of valvular or other disease of the heart, when a considerable impediment exists to the circulation through its chambers, we know that the fibrine of the blood will separate from its other constituents, and form the bodies improperly termed polypi, (which are sometimes so closely interwoven with the carnæ columnæ and chordæ tendineæ as to be with difficulty detached,) which, by closing the orifices, or obstructing the action of the valves, not unfrequently prove the immediate cause of death.

These familiar facts all tend to prove—

1st. That nature herself sets up a process by which, under favourable circumstanccs, the cure of aneurism is effected.

2d. That the mode in which she effects this, always in internal aneurism, and frequently in external aneurism, is by the deposition of the fibrine from the blood in the sac of the aneurism, until it becomes filled.

3d. That the fibrine in such cases is deposited in regular concentric laminæ, the oldest or first formed next the sac, those most recently formed nearest the centre.

4th. That a current of blood through the aneurismal sac is a necessary agent in bringing about this result.

5th. That any obstruction to the current by which its velocity and amount are diminished will accelerate the deposition of fibrine in the aneurismal sac.

6th. That once this process has commenced, if the same agents continue in operation, it will go on until the sac becomes filled, and no longer permits of the entrance of blood.

Writers upon aneurism hitherto appear to have been more intent upon solving unimportant points connected with the distinction between true and false aneurism; or in ingenious speculations as to the comparative frequency of aneurism from dilatation of all the coats of the artery, or from rupture of the internal and middle coats, than in investigating the mode in which a spontaneous cure of the disease takes place. Indeed so little notice is taken of this process in some modern works, that one would suppose the authors were either ignorant of the facts just stated, or looked upon the phenomena as too unimportant to dwell upon.

Before proceeding further, there is a point upon which I wish to make a few remarks. In the details of the cases of aneurism treated by compression which have been published within the last three years, the writers speak of the coagulation of the contents of an aneurismal sac, or of developing a coagulum in it by pressure upon the artery at the cardiac side, as if a coagulum or clot, and the concentric laminæ of fibrine which form in aneurisms, were identical: indeed, from the loose manner of expression adopted, it is sometimes difficult to tell whether the writers are aware of the distinction between them. This is the more remarkable, because the two substances in appearance, colour, and consistence, present a remarkable contrast; the one being soft and loose, of a very dark colour, not deposited in any regular order, and composed of the red globules and fibrine of the blood; the other being solid and firm, of a paler colour, deposited in regular concentric laminæ, and composed of fibrine alone, or with a very small proportion of the red globules. The former is commonly found in the auricles of the heart, and in the large veins which open into them, and is familiar to every body; the latter constitutes the solid matter, which, in greater or less quantity, fills the sac of old aneurisms.

It is obvious, therefore, that the mode in which these two different deposits are formed cannot be the same. To cause the deposition of fibrine in an aneurismal sac, it is essential that a stream of blood should pass through it for a period that will vary according to different circumstances; but its deposition will be promoted or encouraged by diminishing the strength of the current in the artery leading to it, and by lessening the amount of blood which passes through the sac. This, it is easy to understand, can be readily accomplished by compressing the artery at the cardiac side of the aneurism, and the pressure need not be so strong as to occasion very great pain to the patient.

On the other hand, to bring about the coagulation of the contents of an aneurismal sac, the blood must remain at perfect rest for a considerable time; if a current continues to pass through the sac, the blood in it will be replaced by another portion before there is time for its coagulation; for although a coagulum or clot will quickly form when blood is removed from a vein, it is not so easy a matter to bring about its coagulation in an aneurismal sac in a living subject. To effect this, very considerable pressure would be necessary; the compression likewise, it appears to me, would require to be made upon both the cardiac and capillary side of the sac, and very near the latter; while the process will necessarily be so painful that few patients would be willing to submit to it.

It would appear then—

1st. That it is not by the formation of a coagulum in the aneurismal sac that nature effects the cure of the disease, but by the deposition of the fibrine from the blood which circulates through the sac.

2d. That simply diminishing the current will not cause the coagulation of the contents of an aneurism, but it will occasion the deposition of fibrine in the sac."

---

*Lectures on Subjects connected with Clinical Medicine.* By P. M. Latham, M. D., Fellow of the Royal College of Physicians, and Physician to St. Bartholomew's Hospital. Second edition. 8vo. pp, 158. Barrington & Haswell: Philadelphia, 1847.

We are pleased to see a second American edition of the lectures contained in this volume. Few medical writers of our day equal, and none excel Dr. Latham, in clearness and conciseness of expression, aptness of illustration, or justness of the views he inculcates.

The volume before us consists of fifteen lectures on various subjects—all deserving of the greatest consideration. The first is "On the Education of Medical Men," and presents the clearest and soundest view of the subject we have ever read—no sophomore declamation, but sound, common sense reasoning. A short extract or two will show the author's style, and something of his views on this subject.

"There is no subject which at this time more needs the consideration of sober-thinking and reasonable men, than that of professional education.

The subject of education generally is one of great difficulty; yet, strange to say, it is one upon which any body thinks himself qualified to construct a system, and establish rules, and dictate to a whole community; not aware, perhaps, that the wisest men have exercised themselves upon the same, with very doubtful success.

Milton wrote a treatise on education, and so did Locke. And it was in reference to them that Dr. Johnson said, 'Education in England has been in danger of being hurt by two of her greatest men.'

Strange things have been said in jest, or in earnest, concerning the studies necessary to form a physician. Sydenham advised Sir Richard Blackmore to read Don Quixotte. He probably spoke in jest. But it is impossible to read Sydenham and not perceive that his mind did in truth hardly admit any auxiliary to the exercise of its own observation. What he says of anatomy must startle the pains-taking pathologist of modern times, who bestows all his industry in tracing diseases home to the primitive structure in which they are engendered. Anatomy, he has told us, is only fit for a painter. Sydenham might have been *professionally* learned; but there is no evidence in his works that he was so. Perhaps he was offended with the kind of learning then in repute, which tended to make the practice of physic rest chiefly upon authority; or, perhaps, the very structure of his mind was such, that it was really incapable of gaining any thing second-hand which it could gather fresh from the reality; or perhaps he had a noble confidence in that wonderful faculty of observation with which he was endowed, and so resolved to use and to trust it to the uttermost, unaided and unencumbered by any foreign helps.

But Sydenham's education, in so far as it was not professional, was evidently of the best kind. His mind had been early acquainted with a strict, indeed with a scholastic discipline. His book is that of a scholar; I do not mean on account of the language in which it is written, but in its form and structure. Without any apparent artifice, the material facts, and delineations, and histories, arrange themselves all in the right places for being well understood; and they are all so skilfully reasoned upon as to lead you easily and naturally to the conclusions he desired.

Are we then justified, not by the advice of Sydenham, which was probably spoke in jest, but by his example, in concluding that nothing more is required, to fit a man for the safe and successful practice of physic, than a good *general* education, and an industrious use of his own observation in his intercourse with the sick? I think not; and, among many other reasons, especially for the two following:—First, Because you cannot tax the faculty of observation beyond its *common* powers and capacities, you cannot estimate it in the generality by what it was in Sydenham, and look to it for the same results. This would be the same thing as to take measure of all mankind by the proportions of a giant, and make the single wonder of a hundred years the common expectation of every day.

Secondly, Supposing the faculty of observation existed in each of us at its utmost perfection, and enabled each to learn by it all that it is in its own nature capable of teaching; yet there are objects beyond its reach, the knowledge of which has, in the progress of our art, come to be considered as essential to the safe and successful practice of physic as those which lie strictly within its sphere; I mean the knowledge of morbid processes in all their variety, as they affect the structure and functions of our bodies. This knowledge has been derived from other methods of research, from anatomy, from chemistry, from experiment; and you can only gain it either by the use of such methods yourselves, or from the instruction of those who have used them. Sydenham himself could gain it in no other way.

But in our own day, there is little fear that students will be spoiled by the recommendation of their instructors to be content with a scanty knowledge, and trust to their own sagacity for the rest. They are not likely to suffer harm by having Sydenham held up as an example for imitation; the fear is of another kind (and it is well grounded), namely, that many men of the best abilities and good education will be deterred from prosecuting physic as a profession, in consequence of the necessity indiscriminately laid upon all for impossible attainments, of which no example is or can be held forth for their imitation.

After giving a sketch of the requirements insisted on by some persons before a student "can betake himself with effect to the observation of real disease, and hope to acquire a practical skill in treating it," Dr. Latham proceeds:

"Now I am persuaded that there does not exist at this day in the profession an individual who comes up to the standard which (it is implied) all ought to reach. It has been my happiness to know many men in my time who have had enough of attainment to command my highest respect; some who have reached great eminence during their lives, and some who have been thought worthy of monuments since their deaths; yet I have known one, and one only, who came up to the requirements of an introductory lecture which I have read; and that was Dr. Thomas Young. But Dr. Young stood alone among mankind. The most learned and scientific men of his time were struck with wonder at the extent and variety of his knowledge; yet Dr. Young was the only person whom any man now alive ever saw learned and scientific enongh for a physician, according to the Utopian measure of things.

If all medical students had fifteen or twenty years at their disposal, and could dedicate them all to professional education, we might pardon a little innocent declamation in displaying the rich and varied field of knowledge about to be disclosed to them; but even then, sober truth would compel us to confess that the field so pompously displayed far exceeded in extent what the best minds could hope to compass, even in fifteen or twenty years. When, however, we recollect what space of time the majority of men so addressed really can give to their education, the whole affair becomes inexpressibly ludicrous.

Now I do protest, in the name of common sense, against all such proceeding as this. It is all very fine to insist that the eye cannot be understood without the knowledge of optics, nor the circulation without hydraulics, nor the bones and the muscles without mechanics; that metaphysics may have their use in leading us through the functions of the nervous system, and the mysterious connection of mind and matter. It is a truth; and it is a truth also that the whole circle of the sciences is required to comprehend a single particle of matter: but the most solemn truth of all is, *that the life of man is three score years and ten.*

How has it happened that while, in other countries, the medical profession has been exhibited under every imaginable form of ridicule, here, in England, it has been so seldom chosen as a fit thing to laugh at? The truth is, that here no idea of ridicule was ever popularly associated with it; and to have exhibited it as if there were, would have been out of nature and unsuccessful.

A vain, pompous, counterfeit form of knowledge without, and a downright stolid ignorance and incapacity within, made up a precious combination, which, not long ago, was found every where abroad. The mockery and fun that it excited were irresistible and inexhaustible; and Molière and Le Sage made the world ring with laughter at the expense of physic and physicians.

Depend upon it, what all men indiscriminately are told they *ought* to know, all men indiscriminately will soon *pretend* to know, be it never so extravagant; and when every medical man in every town and village throughout England, be he physician, surgeon, or apothecary, shall, in right of his profession, claim the homage due to vast learning and science, there will not be wanting some Molière or Le Sage to hold us all up to the just ridicule of mankind.

Let us take care then what we are about, and beware how we change the character of the English practitioner of physic. He is sound and unpretending, and full of good sense: what he wants is a little more careful, and a somewhat larger instruction in what bears directly upon the practical part of his profession. Give it him (indeed we *are* giving it him), and he will become more trustworthy and more respected every day. But for all that is beyond this, we may recommend it, but we must not insist upon it; we must leave it for each man to pursue according to his leisure, his opportunities, and his capacity, and not exaggerate it into a matter of necessity for all. Where too much is exacted, too little will be learned; excess on the one hand naturally leads to defect on the other.

I know that much disquietude, if not unhappiness, has been felt by students, and especially by the best informed and best disposed, when, at the entrance of their profession, they have been met by obstacles which seem insurmountable. It is the special infirmity of ingenuous minds to reflect with too much anxiety upon their own knowledge; to sit in judgment upon themselves, calculating whether they have made the best of all their opportunities, and wishing, vainly wishing, that their time might come over again, to enable them to supply this omission, or rectify that mistake. By many such, who are at all times too ready to deal hardly with themselves, every exaggerated statement of what they are required to learn is severely felt.

A well-weighed scheme of professional education, sound and practicable, comprehensive yet moderate in its requirements, and adapted to all, besides the many good purposes it would serve, would have the special benefit of satisfying the minds of students themselves that at each step of their progress they are in the right path. Such a scheme we are not likely to have soon. I will not presume to suggest what it ought to be; I would rather endeavour to show you, that, in spite of what you are at present inclined to fear, you may hope to earn a good reputation, to deserve and gain the approbation of mankind as others have deserved and gained it, although you do not possess a perfect literary and philosophical education.

Turn away, then, from the contemplation of this ideal perfection, which can only make you despair, and look to some real examples for your encouragement. But take care that they be *high* examples, and such as no small or ignoble efforts can imitate."

The chapters on "The Doctrine of Symptoms," embrace auscultation and the various physical signs of disease, and ought to be studied by all who aspire to a knowledge of these subjects.

*Half-Yearly Abstract of the Medical Sciences.* By W. H. Ranking, M. D., etc. Assisted by Drs. Guy, Day, Ancell, and Kirkes. No. 5, *January to July*, 1847. Philadelphia, Lindsay and Blakiston.

We had hardly thought of looking for this number until we found it upon our table, so prompt have all those been who are concerned in bringing it out; to the American publishers, in particular, the greatest praise is due, for supplying it to us at so early a date after its appearance in Europe.

In looking over the present number, we have found the same careful selection of matter and admirable arrangement so conspicuous in its predecessors. The "*Reports*," which constitute an important feature of the work, are characterized by great ability and impartiality.

